# Postoperative renal dysfunction and associated perioperative factors among patients undergoing major vascular surgery at Tikur Anbessa Specialized Hospital, Addis Ababa, Ethiopia

**DOI:** 10.1371/journal.pone.0351987

**Published:** 2026-06-22

**Authors:** Mesafint Amare Kebede, Blen Ayele Mamo, Mesfin Sertse Gebremedhin, Mathilda Worku Chala, Siham Faris Isa, Amare Hailu Ashine, Abdullatif Haji-Ababor Abagojam, Abdulhakim Kemal Duressa, Sudi Temam Aman, Sherefudin Hassen Hussen, Merid Lemma Kebede, Kedir Negesso Tukeni

**Affiliations:** 1 Department of Anesthesiology, Tikur Anbessa Specialised Hospital, Addis Ababa, Ethiopia; 2 Department of Anesthesiology, St Paul’s Hospital Millennium Medical College, Addis Ababa, Ethiopia; 3 Department of Clinical Anesthesia, Jimma University, Jimma, Ethiopia; 4 Department of Internal Medicine, Jimma University, Jimma, Ethiopia; 5 Department of General and Laparoscopic Surgery, Jimma University, Jimma, Ethiopia; 6 Department of Orthopedics and Trauma Surgery, Jimma University, Jimma, Ethiopia; 7 Department of Radiology, Jimma University, Jimma, Ethiopia; 8 Department of Internal Medicine and Cardiology, Jimma University, Jimma, Ethiopia; Menzies School of Health Research: Charles Darwin University, AUSTRALIA

## Abstract

**Background:**

Postoperative renal dysfunction (PORD) is a common and serious complication following major vascular surgery and is associated with prolonged hospitalization, renal replacement therapy, and mortality. However, evidences regarding its incidence and associated perioperative factors in resource-limited settings such as Ethiopia remains scarce.

**Objective:**

To determine the incidence, severity distribution, and perioperative factors associated with PORD among patients undergoing major vascular surgery at Tikur Anbessa Specialized Hospital.

**Methods:**

A retrospective cross-sectional study was conducted at Tikur Anbessa Specialized Hospital, Addis Ababa, Ethiopia, between January 2018 and December 2024. A total of 377 adult patients who underwent major vascular surgery were included. Renal outcomes were defined and staged according to the kidney disease: Improving Global Outcomes (KDIGO) criteria. Multivariable logistic regression was performed to identify factors independently associated with PORD. Adjusted odds ratios (AOR) with 95% confidence intervals (CI) were reported.

**Results:**

PORD occurred in 22.5% of patients (95% CI: 18.4–26.9%). Among affected patients, 61.2% had mild dysfunction (Stage 1), 24.7% moderate dysfunction (Stage 2), and 14.1% severe dysfunction (Stage 3). Factors independently associated with PORD included age ≥ 60 years (AOR = 5.99, 95% CI: 3.32–10.80), chronic kidney disease (AOR = 3.49, 95% CI: 1.52–8.03), diabetes mellitus (AOR = 2.37, 95% CI: 1.23–4.57), intraoperative blood loss ≥500 mL (AOR = 4.63, 95% CI: 2.13–10.09), and inadequate urine output (AOR = 2.36, 95% CI: 1.13–4.92).

**Conclusions:**

PORD affected nearly one-quarter of patients undergoing major vascular surgery, with most cases classified as mild dysfunction. Advanced age, chronic kidney disease, diabetes mellitus, excessive intraoperative blood loss, and inadequate urine output were significantly associated with PORD. Enhanced perioperative risk assessment and optimization of comorbidities, and careful intraoperative monitoring may help reduce postoperative renal dysfunction in major vascular surgery patients.

## Background

Major vascular surgery involves procedures on large arteries and veins, including aortic aneurysm repair, carotid endarterectomy, and peripheral bypass grafting, which are performed for life-threatening conditions such as aneurysmal rupture, severe carotid stenosis, and limb-threatening ischemia [1,2]. Globally, postoperative renal dysfunction (PORD) is among the most serious complications following vascular surgery, with reported incidence ranging from 20–30% [2–4]. Although most cases are mild, a substantial proportion progress to moderate or severe renal dysfunction, increasing the risk of dialysis, prolonged hospitalization, and mortality [5,6]. These findings highlight the considerable clinical burden and perioperative significance of PORD worldwide.

The development of PORD is multifactorial and involves preoperative, intraoperative, and postoperative contributors. Important preoperative factors include advanced age, hypertension, diabetes mellitus, chronic kidney disease, cardiovascular disease, and impaired baseline renal function [7,8]. Intraoperative contributors such as hypotension, excessive blood loss, prolonged surgery, fluid imbalance, vasopressor use, and reduced urine output may compromise renal perfusion and exacerbate kidney injury [9]. Postoperative complications, including infection, thrombosis, diuretic use, and prolonged hospital stay, may further worsen renal outcomes [10]. Together, these factors demonstrate that postoperative renal dysfunction arises from a complex interaction of perioperative conditions affecting renal perfusion and systemic hemodynamic stability.

International initiatives, including the kidney disease: Improving Global Outcomes (KDIGO) guidelines, emphasize early recognition of renal injury, perioperative hemodynamic optimization, fluid management, and timely intervention to reduce progression to severe dysfunction [7,8]. In high-resource settings, advanced perioperative monitoring techniques, including continuous cardiac output monitoring and renal perfusion assessment, as well as timely initiation of renal replacement therapy, have improved outcomes among high-risk vascular surgery patients. Despite these advances, implementation of such interventions remains limited in many low- and middle-income countries.

In sub-Saharan Africa, the burden of comorbid conditions associated with postoperative renal dysfunction is increasing. Hypertension and diabetes mellitus have become major public health concerns, while chronic kidney disease remains underdiagnosed and undertreated in many settings [8,10]. Limited access to advanced perioperative monitoring, intensive care support, and dialysis services may increase vulnerability to postoperative renal complications and worsen surgical outcomes across the region.

In Ethiopia, tertiary referral hospitals such as Tikur Anbessa Specialized Hospital (TASH) increasingly perform complex vascular surgical procedures. National surveys estimate that hypertension affects approximately 21% of Ethiopian adults, while diabetes prevalence is around 5%, with both conditions showing increasing trends over the past decade [8,10]. Chronic kidney disease is also increasingly recognized, compounding perioperative risk. Despite expanding surgical capacity, perioperative resources such as advanced hemodynamic monitoring and renal support services remain constrained, potentially increasing the likelihood of postoperative renal dysfunction among high-risk surgical patients.

Previous Ethiopian studies have largely focused on isolated vascular procedures, such as carotid endarterectomy or aneurysm repair, without comprehensively evaluating the incidence, severity, distribution, and perioperative factors associated with postoperative renal dysfunction across the broader spectrum of vascular surgeries [8,9]. Consequently, evidence regarding the burden and perioperative factors associated with PORD among Ethiopian vascular surgery patients remains limited. Addressing this gap is important for improving perioperative risk stratification, optimizing intraoperative management, and guiding preventive strategies in resource-constrained settings.

Therefore, this study aimed to determine the incidence, severity distribution, and perioperative factors associated with postoperative renal dysfunction among patients undergoing major vascular surgery at Tikur Anbessa Specialized Hospital, Addis Ababa, Ethiopia.

## Methods

### Study design and setting

We conducted a retrospective cohort study at Tikur Anbessa Specialized Hospital (TASH), Addis Ababa, Ethiopia, between January 2018 and December 2024. TASH is Ethiopia’s largest tertiary referral and teaching hospital, providing a wide range of surgical interventions, including general, orthopedic, cardiothoracic, and vascular surgery. Because all eligible patients during the study period were included (n = 377), the reported incidence represents the observed population proportion rather than a sample estimate; however, binomial confidence intervals are provided for transparency.

### Eligibility criteria

Adult patients (≥18 years) who underwent major vascular surgery during the study period were included if they had complete perioperative records, documented baseline and follow‑up serum creatinine, and hospitalization for at least 48 hours postoperatively. Patients with prior dialysis, end‑stage renal disease, or incomplete records were excluded.

### Sample size and sampling procedure

The initial sample size was calculated using the single population proportion formula, assuming a 50% estimated incidence of postoperative renal dysfunction (PORD), a 95% confidence level, and a 5% margin of error. The assumption of 50% was selected because no prior Ethiopian study had reported the incidence of PORD among major vascular surgery patients, and this proportion provides the maximum sample size for adequate precision.

The calculated sample size was 384 patients. However, because the total number of eligible patients who underwent major vascular surgery at Tikur Anbessa Specialized Hospital during the study period (January 2018–December 2024) was 377, all eligible patients were included using a census sampling approach. Therefore, no additional sampling procedure was performed.

### Operational definitions

- **Major vascular surgery:** Procedures involving large arteries or veins, including aortic aneurysm repair, carotid endarterectomy, femoral-popliteal bypass, thrombectomy, and interposition grafts [1,2].
- **Postoperative renal dysfunction (PORD):** Defined according to KDIGO criteria as any of the following:

- ≥50% increase in serum creatinine from baseline,
- increase in serum creatinine by ≥0.3 mg/dL within 48 hours,
- increase to ≥1.5 times baseline within 7 days,
- urine output <0.5 mL/kg/hr for ≥6 hours [7,8].

Severity was classified using KDIGO staging criteria as Stage 1 (mild), Stage 2 (moderate), and Stage 3 (severe) [7,8]. Patients meeting multiple criteria were assigned the highest stage reached during the first 7 postoperative days.

### Data collection tool and diagnosis

Data were extracted from inpatient medical records using a structured checklist developed for this study, capturing sociodemographic, preoperative, intraoperative, and postoperative variables. Diagnoses were confirmed by senior specialists and cross‑checked against laboratory results. Medical chart data were merged with laboratory investigations through patient identifiers to ensure consistency.

### Data quality assurance

To ensure data quality, a structured data extraction checklist was developed based on the study objectives and relevant literature. Data collectors and supervisors received orientation regarding the objectives of the study, operational definitions, and procedures for data extraction before data collection began. Patient records were reviewed systematically, and extracted data were cross-checked against laboratory reports and operative records to ensure consistency and completeness. Double data entry was performed using EpiData software to minimize entry errors, and regular supervision was conducted throughout the data collection process. Incomplete records and inconsistent entries were verified using source documents whenever possible.

Laboratory results were obtained from standardized hospital laboratory records, where routine internal quality control procedures and equipment calibration were performed according to institutional protocols. To improve diagnostic reliability, only cases documented and confirmed by senior specialists were included in the analysis. Data cleaning, coding, and validation were completed before analysis to ensure accuracy and consistency of the dataset.

### Data analysis

Data were analyzed using SPSS version 27. Bivariate logistic regression identified candidate variables, followed by multivariable logistic regression to determine significantly associated factors. Results are presented as adjusted odds ratios (AOR) with 95% confidence intervals (CI) to quantify the strength and precision of association, and p‑values to indicate statistical significance. Model fitness was assessed using the Hosmer–Lemeshow test (p > 0.05 indicating good fit). Multicollinearity was checked using variance inflation factors (VIF < 10). Before the multivariable logistic regression analysis, assumptions for logistic regression were assessed. The adequacy of the sample size and outcome events was evaluated using the events-per-variable principle to ensure model stability. Variables with p < 0.25 in bivariable analysis were entered into multivariable logistic regression. Multicollinearity was assessed using VIF (<10 acceptable), and model fitness was evaluated using the Hosmer–Lemeshow test.

### Temporal trends

We examined diagnoses and surgical interventions across the study period (2018–2024). Incidence of PORD remained relatively stable (20–23%), with minor fluctuations. No major changes in surgical protocols or international recommendations were adopted at TASH during this period, which explains the consistency of outcomes. Postoperative renal dysfunction (PORD) was classified by KDIGO 2012 acute kidney injury staging and mapped to plain‑language severity categories for clarity. We used baseline serum creatinine recorded preoperatively and postoperative creatinine and urine‑output measurements to assign KDIGO stages as follows: **Stage 1 (mild)** — increase in serum creatinine ≥0.3 mg/dL within 48 hours or 1.5–1.9 × baseline, or urine output <0.5 mL/kg/hr for 6–12 hours; **Stage 2 (moderate)** — serum creatinine 2.0–2.9 × baseline or urine output <0.5 mL/kg/hr for ≥12 hours; **Stage 3 (severe)** — serum creatinine ≥3.0 × baseline or absolute serum creatinine ≥4.0 mg/dL, initiation of renal replacement therapy, or urine output <0.3 mL/kg/hr for ≥24 hours or anuria for ≥12 hours. Patients meeting any KDIGO criterion were assigned the highest stage reached during the first 7 postoperative days. We report both KDIGO stage and the corresponding plain‑language category (mild/moderate/severe). Where baseline creatinine was missing, we used the most recent preoperative value recorded within 3 months; cases without any preoperative creatinine were excluded from severity analysis.

### Ethical approval

Ethical clearance was obtained from the Institutional Review Board of Addis Ababa University, College of Health Sciences. Informed consent was waived due to the retrospective nature of the study. The study adhered to the principles of the Declaration of Helsinki. Informed consent was waived due to the retrospective nature of the study; patient confidentiality was maintained by anonymizing data during extraction and analysis.

## Results

### Baseline and perioperative characteristics

A total of 377 patients who underwent major vascular surgery between January 2018 and December 2024 were included in the study. Of these, 196 (52.0%) were male, and 89 (23.6%) were aged ≥60 years. Hypertension was present in 165 (43.8%) patients, diabetes mellitus in 66 (17.5%), and chronic kidney disease in 34 (9.0%). Most procedures were elective (55.7%) and performed under general anesthesia (66.8%). Intraoperative blood loss ≥500 mL occurred in 255 (67.6%) patients, while inadequate urine output was observed in 88 (23.3%) cases (Table 1).

**Table 1:** Baseline and perioperative characteristics of patients undergoing major vascular surgery according to postoperative renal dysfunction (PORD) status at Tikur Anbessa Specialized Hospital, Addis Ababa, Ethiopia, 2018–2024 (n = 377).

Variable	Category	PORD Yes n (%)	PORD No n (%)	Total n (%)	p-value
**Sex**	Male	47 (24.0)	149 (76.0)	196 (52.0)	0.041
	Female	38 (21.0)	143 (79.0)	181 (48.0)	
**Age group**	<60 years	38 (13.2)	250 (86.8)	288 (76.4)	<0.001
	≥60 years	47 (52.8)	42 (47.2)	89 (23.6)	
**Hypertension**	Yes	52 (31.5)	113 (68.5)	165 (43.8)	0.002
	No	33 (15.6)	179 (84.4)	212 (56.2)	
**Diabetes**	Yes	29 (43.9)	37 (56.1)	66 (17.5)	<0.001
	No	56 (18.0)	255 (82.0)	311 (82.5)	
**CKD**	Yes	21 (61.8)	13 (38.2)	34 (9.0)	<0.001
	No	64 (18.7)	279 (81.3)	343 (91.0)	
**ASA Physical Status**	ASA I–II	18 (19.6)	74 (80.4)	92 (24.4)	0.003
	ASA III–IV	66 (24.3)	206 (75.7)	272 (72.1)	
	ASA V	1 (33.3)	2 (66.7)	3 (0.8)	
**Type of surgery**	Elective	39 (18.6)	171 (81.4)	210 (55.7)	0.018
	Emergency	46 (27.5)	121 (72.5)	167 (44.3)	
**Anesthesia**	General anesthesia	66 (26.2)	186 (73.8)	252 (66.8)	0.022
	Neuraxial/regional	19 (15.2)	106 (84.8)	125 (33.2)	
**Intraoperative hypotension**	Yes	55 (31.1)	122 (68.9)	177 (46.9)	<0.001
	No	30 (15.0)	170 (85.0)	200 (53.1)	
**Blood loss**	<500 mL	10 (8.2)	112 (91.8)	122 (32.4)	<0.001
	≥500 mL	75 (29.4)	180 (70.6)	255 (67.6)	
**Urine output**	Adequate	23 (8.0)	266 (92.0)	289 (76.7)	<0.001
	Inadequate	62 (70.5)	26 (29.5)	88 (23.3)	
**Postoperative vasopressor use**	Yes	24 (60.0)	16 (40.0)	40 (10.6)	<0.001
	No	61 (18.1)	276 (81.9)	337 (89.4)	
**Length of hospital stay**	≤10 days	48 (17.1)	233 (82.9)	281 (74.5)	0.001
	>10 days	37 (38.5)	59 (61.5)	96 (25.5)	

**NB**: *Percentages were calculated within each category. Comparisons between groups were performed using the chi-square test or Fisher’s exact test where appropriate. ASA = American Society of Anesthesiologists physical status classification; PORD = postoperative renal dysfunction*.

### Incidence and severity distribution of postoperative renal dysfunction

**Fig 1:**
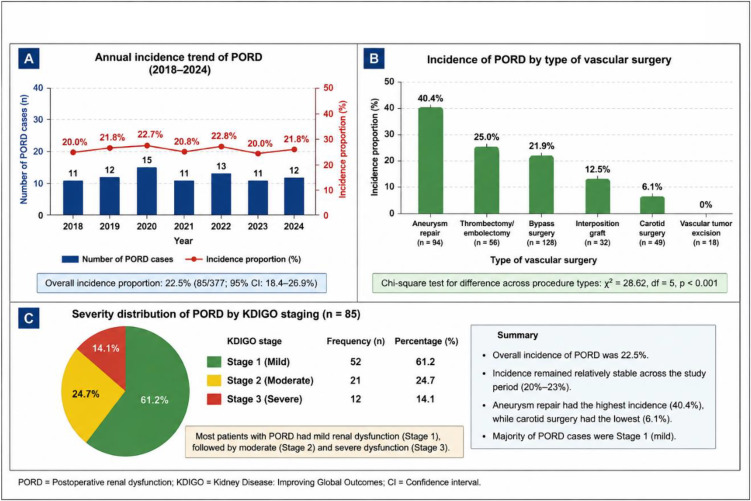
Incidence and severity distribution of postoperative renal dysfunction (PORD) among patients undergoing major vascular surgery at Tikur Anbessa Specialized Hospital, Addis Ababa, Ethiopia, 2018–2024. **Panel A** illustrates annual incidence trends of postoperative renal dysfunction across the study period, demonstrating relatively stable incidence proportions ranging from 20% to 23%. **Panel B** presents procedure-specific incidence proportions, showing the highest incidence following aneurysm repair procedures and the lowest incidence following carotid surgeries. **Panel C** demonstrates the severity distribution of postoperative renal dysfunction according to KDIGO staging criteria, where most cases were classified as Stage 1 (mild dysfunction), followed by Stage 2 (moderate dysfunction) and Stage 3 (severe dysfunction).

Among the 377 patients included in the study, 85 developed postoperative renal dysfunction (PORD), yielding an incidence proportion of 22.5% (95% CI: 18.4–26.9%). The incidence of PORD remained relatively stable throughout the study period, ranging from 20% to 23%, with minor annual fluctuations. Severity assessment based on kidney disease: Improving Global Outcomes (KDIGO) staging demonstrated that most affected patients had mild renal dysfunction. Of the 85 patients with PORD, 52 (61.2%) were classified as Stage 1 (mild), 21 (24.7%) as Stage 2 (moderate), and 12 (14.1%) as Stage 3 (severe). Procedure-specific analysis showed variability in the incidence of PORD across vascular surgery types. The highest incidence was observed following aneurysm repair procedures (40.4%), followed by thrombectomies (25.0%) and bypass surgeries (21.9%). Lower incidence proportions were observed among patients undergoing carotid surgeries (6.1%) and interposition graft procedures (12.5%). No cases of postoperative renal dysfunction were identified among patients undergoing vascular tumor excision procedures (Fig 1).

### Cross-tabulation of perioperative characteristics according to PORD status

Comparative analysis demonstrated significant differences in perioperative characteristics between patients who developed postoperative renal dysfunction (PORD) and those who did not (Table 1). The incidence of PORD was substantially higher among patients aged ≥60 years compared with younger patients (52.8% vs 13.2%, p < 0.001). Similarly, patients with hypertension, diabetes mellitus, and chronic kidney disease experienced significantly higher incidence proportions of postoperative renal dysfunction.

Perioperative surgical factors were also strongly associated with PORD occurrence. Patients undergoing emergency surgery had a higher incidence of postoperative renal dysfunction compared with those undergoing elective procedures (27.5% vs 18.6%, p = 0.018). Likewise, PORD occurred more frequently among patients who underwent surgery under general anesthesia compared with neuraxial or regional anesthesia (26.2% vs 15.2%, p = 0.022).

Intraoperative hemodynamic and renal perfusion variables demonstrated particularly strong associations with postoperative renal dysfunction. Patients with intraoperative hypotension had more than double the incidence of PORD compared with those without hypotension (31.1% vs 15.0%, p < 0.001). Similarly, excessive intraoperative blood loss (≥500 mL) and inadequate urine output were significantly more common among patients who developed postoperative renal dysfunction. The incidence of PORD among patients with inadequate urine output reached 70.5%, compared with 8.0% among patients with adequate urine output (p < 0.001).

Postoperative vasopressor use and prolonged hospital stay were also significantly associated with PORD. Patients requiring postoperative vasopressors experienced a markedly higher incidence of renal dysfunction compared with those who did not require vasopressor support (60.0% vs 18.1%, p < 0.001). In addition, patients hospitalized for more than 10 days had a significantly higher incidence of postoperative renal dysfunction than those with shorter hospital stays (38.5% vs 17.1%, p = 0.001).

### Factors independently associated with postoperative renal dysfunction

Variables with p < 0.25 in bivariable analysis were entered into the multivariable logistic regression model. After adjustment for potential confounders, advanced age, chronic kidney disease, diabetes mellitus, excessive intraoperative blood loss, and inadequate urine output remained independently associated with postoperative renal dysfunction (Table 2). Patients aged ≥60 years had nearly sixfold higher odds of developing postoperative renal dysfunction compared with younger patients (AOR = 5.99, 95% CI: 3.32–10.80). Similarly, patients with chronic kidney disease had more than threefold increased odds of postoperative renal dysfunction (AOR = 3.49, 95% CI: 1.52–8.03), while diabetes mellitus was associated with more than twofold higher odds of renal dysfunction (AOR = 2.37, 95% CI: 1.23–4.57). Among intraoperative variables, blood loss ≥500 mL was strongly associated with postoperative renal dysfunction, increasing the odds by more than fourfold (AOR = 4.63, 95% CI: 2.13–10.09). Inadequate urine output during surgery was also independently associated with postoperative renal dysfunction (AOR = 2.36, 95% CI: 1.13–4.92).

**Table 2:** Multivariable logistic regression analysis of factors independently associated with postoperative renal dysfunction among patients undergoing major vascular surgery at Tikur Anbessa Specialized Hospital, Addis Ababa, Ethiopia, 2018–2024 (n = 377).

Variable	Category	COR (95% CI)	AOR (95% CI)
**Age group**	<60 years	1.00	1.00
	≥60 years	7.36 (4.33–12.51)	5.99 (3.32–10.80)
**Sex**	Female	1.00	1.00
	Male	1.19 (0.74–1.92)	1.12 (0.64–1.95)
**Hypertension**	No	1.00	1.00
	Yes	2.49 (1.51–4.10)	1.41 (0.78–2.56)
**Diabetes mellitus**	No	1.00	1.00
	Yes	3.57 (2.01–6.32)	2.37 (1.23–4.57)
**Chronic kidney disease**	No	1.00	1.00
	Yes	7.05 (3.34–14.88)	3.49 (1.52–8.03)
**Type of surgery**	Elective	1.00	1.00
	Emergency	1.67 (1.02–2.72)	1.28 (0.71–2.31)
**General anesthesia**	No	1.00	1.00
	Yes	1.98 (1.13–3.46)	1.24 (0.65–2.36)
**Intraoperative hypotension**	No	1.00	1.00
	Yes	2.56 (1.54–4.25)	1.44 (0.79–2.61)
**Blood loss**	<500 mL	1.00	1.00
	≥500 mL	4.67 (2.33–9.38)	4.63 (2.13–10.09)
**Urine output**	Adequate	1.00	1.00
	Inadequate	27.60 (14.79–51.52)	2.36 (1.13–4.92)
**Postoperative vasopressor use**	No	1.00	1.00
	Yes	6.79 (3.34–13.79)	1.72 (0.74–3.98)

NB: COR = crude odds ratio; AOR = adjusted odds ratio; CI = confidence interval. Variables with p < 0.25 in bivariable analysis were included in the multivariable logistic regression model. Model fitness was assessed using the Hosmer–Lemeshow goodness-of-fit test.

### Distribution of major perioperative characteristics according to postoperative renal dysfunction (PORD) status among patients undergoing major vascular surgery at Tikur Anbessa Specialized Hospital, Addis Ababa, Ethiopia

**Fig 2:**
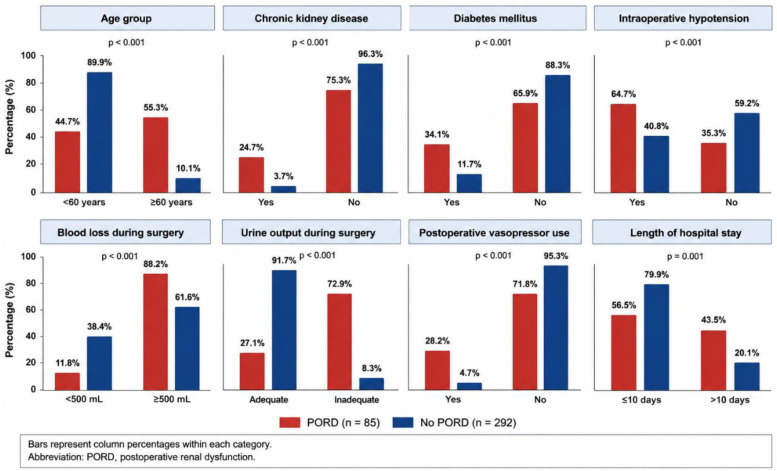
Distribution of major perioperative characteristics associated with postoperative renal dysfunction among patients undergoing major vascular surgery at Tikur Anbessa Specialized Hospital, Addis Ababa, Ethiopia, 2018–2024. Abbreviations: PORD = postoperative renal dysfunction.

Fig 2 demonstrates notable differences in perioperative characteristics between patients who developed postoperative renal dysfunction (PORD) and those who did not. Higher proportions of PORD were observed among patients aged ≥60 years, those with chronic kidney disease and diabetes mellitus, and patients who experienced intraoperative hypotension, excessive blood loss, inadequate urine output, or postoperative vasopressor use. In particular, inadequate urine output and blood loss ≥500 mL showed the biggest differences between the two groups. Additionally, patients with prolonged hospital stay (>10 days) had a substantially higher proportion of postoperative renal dysfunction compared with those hospitalized for shorter durations. Overall, the figure highlights the important contribution of preoperative comorbidities and intraoperative hemodynamic instability to the development of postoperative renal dysfunction following major vascular surgery.

## Discussion

This study assessed the incidence, severity distribution, and perioperative factors associated with postoperative renal dysfunction (PORD) among patients undergoing major vascular surgery at Tikur Anbessa Specialized Hospital. The overall incidence proportion of PORD was 22.5%, with most cases classified as mild dysfunction according to the Kidney Disease: Improving Global Outcomes (KDIGO) staging criteria. Advanced age, chronic kidney disease, diabetes mellitus, excessive intraoperative blood loss, and inadequate urine output were independently associated with postoperative renal dysfunction. These findings highlight the substantial burden of perioperative renal complications among vascular surgery patients in resource-constrained settings.

The observed incidence proportion of postoperative renal dysfunction in this study is consistent with reports from international vascular surgery cohorts, where incidence estimates commonly range from 20% to 30% depending on surgical complexity, baseline patient risk, and diagnostic criteria used [5,6]. The relatively high incidence observed in the present study may reflect the complex nature of major vascular surgery, which frequently involves substantial hemodynamic fluctuations, prolonged operative duration, blood loss, and impaired renal perfusion [7,8]. In addition, limited access to advanced perioperative hemodynamic monitoring and renal support services in low-resource settings may contribute to increased vulnerability to postoperative renal complications.

Most patients with postoperative renal dysfunction in this study were classified as KDIGO Stage 1, indicating predominantly mild renal impairment. Similar severity distributions have been reported in previous studies, where mild postoperative renal dysfunction accounted for the majority of cases following major vascular surgery [5,6]. Although mild dysfunction may appear clinically less severe, even small postoperative changes in renal function have been associated with prolonged hospitalization, increased healthcare costs, and higher risk of subsequent chronic kidney disease and mortality [5,6]. Therefore, early identification and intervention remain clinically important even in apparently mild cases.

Advanced age was strongly associated with postoperative renal dysfunction in the current study. Patients aged 60 years or older had nearly sixfold increased odds of developing postoperative renal dysfunction compared with younger patients. This finding is consistent with previous studies demonstrating increased susceptibility to perioperative renal injury among older surgical patients [7,8]. Age-related decline in renal reserve, vascular stiffness, impaired autoregulatory capacity, and increased prevalence of comorbid conditions may contribute to reduced tolerance to perioperative hemodynamic stress and renal hypoperfusion [8,10].

Chronic kidney disease and diabetes mellitus were also independently associated with postoperative renal dysfunction. Patients with pre-existing chronic kidney disease had substantially higher odds of developing postoperative renal dysfunction, consistent with findings from previous vascular surgery studies [5,6]. Reduced nephron reserve and impaired renal adaptive capacity likely increase susceptibility to perioperative renal ischemia and nephrotoxic exposure. Similarly, diabetes mellitus was associated with increased odds of postoperative renal dysfunction, possibly due to underlying microvascular disease, endothelial dysfunction, and chronic inflammatory changes affecting renal perfusion and recovery [8,10].

Among intraoperative variables, excessive blood loss and inadequate urine output demonstrated strong independent associations with postoperative renal dysfunction. Significant blood loss may reduce effective circulating volume and compromise renal perfusion, thereby increasing the risk of ischemic renal injury [7,8]. Likewise, inadequate urine output during surgery likely reflects impaired renal perfusion and evolving intraoperative hemodynamic instability. These findings emphasize the importance of vigilant perioperative fluid management, hemodynamic optimization, and close renal monitoring during major vascular procedures.

The findings of this study have important clinical implications for perioperative care in resource-constrained settings. Early identification of high-risk patients, optimization of pre-existing comorbidities, careful intraoperative hemodynamic monitoring, and prompt correction of blood loss and fluid imbalance may help reduce postoperative renal complications among vascular surgery patients.

This study has several strengths. First, it provides one of the few comprehensive assessments of postoperative renal dysfunction among major vascular surgery patients in Ethiopia and sub-Saharan Africa. Second, the study included a relatively large seven-year cohort of vascular surgery patients managed at the country’s largest tertiary referral center, improving the clinical relevance of the findings. Third, postoperative renal dysfunction was defined and staged using standardized KDIGO criteria, enhancing diagnostic consistency and comparability with international literature. Finally, the study evaluated multiple perioperative demographic, clinical, and intraoperative variables using multivariable logistic regression analysis to identify factors independently associated with postoperative renal dysfunction.

In conclusion, postoperative renal dysfunction affected nearly one-quarter of patients undergoing major vascular surgery at Tikur Anbessa Specialized Hospital, with most cases classified as mild dysfunction. ‌‌Advanced age, chronic kidney disease, diabetes mellitus, excessive intraoperative blood loss, and inadequate urine output were independently associated with postoperative renal dysfunction. Strengthening perioperative risk stratification, optimizing comorbidity management, and improving intraoperative hemodynamic monitoring may help reduce postoperative renal complications in vascular surgery patients.

## Limitations of the study

This study is a retrospective cross‑sectional analysis and therefore reports factors associated with postoperative renal dysfunction rather than causal determinants; temporality and causation cannot be established. The single‑center design at Tikur Anbessa Specialized Hospital limits generalizability to other settings with different case‑mixes or perioperative resources. In addition, other organ‑specific injuries (for example, cardiac ischemia, hepatic injury, or limb ischemia) were not systematically assessed because documentation was inconsistent across charts; therefore, the study focuses on renal outcomes where baseline and follow‑up creatinine and urine‑output data were reliably available. Finally, missing baseline creatinine in a small subset required exclusion from severity analysis, which may bias severity estimates.

## Conclusion

Postoperative renal dysfunction was common among patients undergoing major vascular surgery, particularly among older patients and those with chronic kidney disease, diabetes mellitus, excessive intraoperative blood loss, and inadequate urine output. Strengthening perioperative risk assessment and hemodynamic optimization may reduce postoperative renal complications.

## Supporting information

S1 Checklist Structured data extraction tool used for collection of socio‑demographic, preoperative, intraoperative, and postoperative variables. (DOCX)

S2 Table Detailed distribution of comorbidities and ASA physical status classification among study participants. (DOCX)

S3 Table Expanded intraoperative parameters including anesthesia type, fluid management, and hemodynamic complications. (DOCX)

S4 Table Postoperative outcomes including complications, interventions, and length of hospital stay. (DOCX)
