# Supplementary material for: Postoperative renal dysfunction and associated perioperative factors among patients undergoing major vascular surgery at Tikur Anbessa Specialized Hospital, Addis Ababa, Ethiopia
Source: PLoS One. 2026 Jun 22;21(6):e0351987. doi: 10.1371/journal.pone.0351987 (PMC13286180; doi:10.1371/journal.pone.0351987)
Supplement: S1 Checklist — (DOCX) [file pone.0351987.s001.docx]

**S1 Checklist.**

**Structured Data Collection Questionnaire**

This checklist was used during retrospective chart review to extract perioperative data for patients undergoing major vascular surgery at Tikur Anbessa Specialized Hospital (2018–2024).

**Sections and Variables Captured:**

1. **Socio‑Demographic Characteristics**
   - Patient ID (coded)
   - Age (years)
   - Sex (male/female)
   - Residence (urban/rural)
2. **Preoperative Factors**
   - ASA physical status classification
   - History of chronic kidney disease (yes/no)
   - History of diabetes mellitus (yes/no)
   - History of hypertension (yes/no)
   - Cardiovascular disease/heart failure (yes/no)
   - Prior vascular surgery (yes/no)
   - Preoperative diuretic use (yes/no)
3. **Intraoperative Parameters**
   - Type of surgery (aneurysm repair, bypass, thrombectomy, carotid surgery, interposition graft, tumor excision)
   - Elective vs emergency procedure
   - Type of anesthesia (general, neuraxial, combined, regional converted to general, peripheral block)
   - Duration of surgery (<2 hrs, 2–4 hrs, >4 hrs)
   - Fluid management (crystalloids only / crystalloids + colloids)
   - Intraoperative complications (hypotension, hypertension, bradycardia, tachycardia, other)
   - Use of vasopressors/inotropes (yes/no)
   - Estimated blood loss (<500 mL / ≥500 mL)
   - Intraoperative urine output (adequate ≥0.5 mL/kg/hr / inadequate <0.5 mL/kg/hr)
4. **Postoperative Outcomes**
   - Use of diuretics (yes/no)
   - Use of vasopressors/inotropes (yes/no)
   - Blood transfusion (yes/no)
   - Postoperative complications (infection, thrombosis, bleeding, fluid collection, other)
   - Length of hospital stay (<5 days, 5–10 days, >10 days)
   - Postoperative urine output adequacy (adequate/inadequate)
5. **Renal Function Assessment (Outcome Variable)**
   - Serum creatinine baseline (mg/dL)
   - Postoperative serum creatinine (mg/dL)
   - Change in creatinine (≥0.3 mg/dL within 48 hrs / ≥50% increase from baseline / ≥1.5× baseline within 7 days)
   - Estimated GFR (mL/min/1.73 m²)
   - Urine output (<0.5 mL/kg/hr for ≥6 hrs)
