# Supplementary material for: Postoperative renal dysfunction and associated perioperative factors among patients undergoing major vascular surgery at Tikur Anbessa Specialized Hospital, Addis Ababa, Ethiopia
Source: PLoS One. 2026 Jun 22;21(6):e0351987. doi: 10.1371/journal.pone.0351987 (PMC13286180; doi:10.1371/journal.pone.0351987)
Supplement: S2 Table — (DOCX) [file pone.0351987.s002.docx]

**S2 Table.**

Expanded socio‑demographic and preoperative characteristics of study participants. Includes age distribution, sex, ASA physical status classification, comorbidities (hypertension, diabetes mellitus, chronic kidney disease, cardiovascular disease), and prior vascular surgery history.

**S2 Table.**

**Expanded Socio‑Demographic and Preoperative Characteristics of Study Participants (n = 377)**

| **Variable** | **Category** | **Frequency (n)** | **Percentage (%)** |
| --- | --- | --- | --- |
| **Sex** | Male | 196 | 52.0 |
|  | Female | 181 | 48.0 |
| **Age group** | < 60 years | 288 | 76.4 |
|  | ≥ 60 years | 89 | 23.6 |
| **ASA Physical Status** | ASA I | 9 | 2.4 |
|  | ASA II | 74 | 19.6 |
|  | ASA III | 192 | 50.9 |
|  | ASA IV | 99 | 26.3 |
|  | ASA V | 3 | 0.8 |
| **Comorbidities** | Hypertension | 165 | 43.8 |
|  | Diabetes mellitus | 66 | 17.5 |
|  | Chronic kidney disease | 34 | 9.0 |
|  | Cardiovascular disease/heart failure | 112 | 29.7 |
| **Prior vascular surgery** | Yes | 89 | 23.6 |
|  | No | 288 | 76.4 |
| **Preoperative diuretic use** | Yes | 46 | 12.2 |
|  | No | 331 | 87.8 |

**Footnote:** Percentages are calculated from the total study population (n = 377). ASA = American Society of Anesthesiologists physical status classification.
