# Supplementary material for: Postoperative renal dysfunction and associated perioperative factors among patients undergoing major vascular surgery at Tikur Anbessa Specialized Hospital, Addis Ababa, Ethiopia
Source: PLoS One. 2026 Jun 22;21(6):e0351987. doi: 10.1371/journal.pone.0351987 (PMC13286180; doi:10.1371/journal.pone.0351987)
Supplement: S3 Table — (DOCX) [file pone.0351987.s003.docx]

**S3 Table.**

Detailed intraoperative characteristics of patients undergoing major vascular surgery. Variables include type of surgery (aneurysm repair, bypass, thrombectomy, carotid surgery, interposition graft, tumor excision), anesthesia techniques, duration of surgery, intraoperative fluid therapy, blood loss categories, hemodynamic complications, and vasopressor/inotrope use.

**S3 Table.**

**Expanded Intraoperative Characteristics of Patients Undergoing Major Vascular Surgery (n = 377)**

| **Variable** | **Category** | **Frequency (n)** | **Percentage (%)** |
| --- | --- | --- | --- |
| **Type of Surgery** | Elective | 210 | 55.7 |
|  | Emergency | 167 | 44.3 |
| **Type of Vascular Surgery** | Aneurysm repair | 57 | 15.1 |
|  | Bypasses (aortofemoral, aortiliac, femoropopliteal, others) | 128 | 33.9 |
|  | Carotid surgery (endarterectomy, others) | 49 | 13.0 |
|  | Major vessel interposition grafts | 21 | 5.6 |
|  | Thrombectomies | 108 | 28.6 |
|  | Vascular tumor excisions | 14 | 3.7 |
| **Type of Anesthesia** | General anesthesia | 252 | 66.8 |
|  | Neuraxial (spinal or epidural) | 86 | 22.8 |
|  | Combined spinal & epidural | 12 | 3.2 |
|  | Peripheral nerve block | 9 | 2.4 |
|  | Regional converted to general | 18 | 4.8 |
| **Duration of Surgery (hours)** | < 2 | 11 | 3.0 |
|  | 2–4 | 187 | 49.6 |
|  | > 4 | 179 | 47.5 |
| **Intraoperative Fluid Management** | Crystalloids only | 264 | 70.0 |
|  | Crystalloids + colloids | 113 | 30.0 |
| **Intraoperative Complications** | Hypotension | 177 | 46.9 |
|  | Hypertension | 29 | 7.7 |
|  | Bradycardia | 7 | 1.8 |
|  | Other (unspecified) | 150 | 39.8 |
| **Use of Vasopressors/Inotropes** | Yes | 153 | 40.6 |
|  | No | 224 | 59.4 |
| **Estimated Blood Loss (mL)** | < 500 | 122 | 32.4 |
|  | ≥ 500 | 255 | 67.6 |
| **Intraoperative Urine Output** | Adequate (≥0.5 mL/kg/hr) | 289 | 76.7 |
|  | Inadequate (<0.5 mL/kg/hr) | 88 | 23.3 |

**Footnote:** Percentages are calculated from the total study population (n = 377). Blood loss categories are defined as <500 mL and ≥500 mL. Urine output adequacy assessed using KDIGO criteria.
