# Supplementary material for: Postoperative renal dysfunction and associated perioperative factors among patients undergoing major vascular surgery at Tikur Anbessa Specialized Hospital, Addis Ababa, Ethiopia
Source: PLoS One. 2026 Jun 22;21(6):e0351987. doi: 10.1371/journal.pone.0351987 (PMC13286180; doi:10.1371/journal.pone.0351987)
Supplement: S4 Table — (DOCX) [file pone.0351987.s004.docx]

**S4 Table.**

Postoperative outcomes and complications. Includes diuretic and vasopressor use, transfusion requirements, postoperative complications (infection, thrombosis, bleeding, fluid collections), length of hospital stay, and adequacy of urine output.

**S4 Table.**

**Expanded Postoperative Outcomes and Complications of Patients Undergoing Major Vascular Surgery (n = 377)**

| **Variable** | **Category** | **Frequency (n)** | **Percentage (%)** |
| --- | --- | --- | --- |
| **Use of Diuretics** | Yes | 79 | 21.0 |
|  | No | 298 | 79.0 |
| **Use of Vasopressors/Inotropes** | Yes | 40 | 10.6 |
|  | No | 337 | 89.4 |
| **Blood Transfusion** | Yes | 35 | 9.3 |
|  | No | 342 | 90.7 |
| **Postoperative Complications** | Infection | 52 | 13.8 |
|  | Thrombosis | 63 | 16.7 |
|  | Bleeding | 8 | 2.1 |
|  | Fluid collection | 8 | 2.1 |
|  | Other (unspecified) | 256 | 67.9 |
| **Length of Hospital Stay** | < 5 days | 50 | 13.3 |
|  | 5–10 days | 230 | 61.0 |
|  | > 10 days | 97 | 25.7 |
| **Postoperative Urine Output Adequacy** | Adequate (≥0.5 mL/kg/hr) | 289 | 76.7 |
|  | Inadequate (<0.5 mL/kg/hr) | 88 | 23.3 |

**Footnote:** Percentages are calculated from the total study population (n = 377). Urine output adequacy assessed using KDIGO criteria.
